# Recurrence of skin and soft tissue infections: identifying risk factors and treatment strategies

**DOI:** 10.1097/QCO.0000000000001096

**Published:** 2025-02-19

**Authors:** Alice Toschi, Maddalena Giannella, Pierluigi Viale

**Affiliations:** a Department of Medical and Surgical Sciences, Alma Mater Studiorum University of Bologna; b Infectious Diseases Unit, Department for Integrated Infectious Risk Management, IRCCS Azienda Ospedaliero-Universitaria di Bologna, Bologna, Italy

**Keywords:** antibiotic prophylaxis, methicillin-resistant *Staphylococcus aureus*, outpatient parenteral antibiotic therapy, recurrent infections, skin and soft tissue infection

## Abstract

**Purpose of review:**

Recurrent skin and soft tissue infections (RSSTIs) are challenging for the clinicians due to morbidity and healthcare-related costs. Here, we review updates on risk factors and management.

**Recent findings:**

RSSTIs rates range between 7 and 45%. Local and systemic conditions can favour RSSTIs, with comorbidities such as obesity, diabetes, cancer and immunosuppressive disease becoming increasingly relevant. *Streptococcus spp*. and *Staphylococcus aures* (including methicillin resistant, MRSA) are the leading causative pathogens of RSSTIs, but also Gram-negative bacteria and polymicrobial infection should be considered. To prevent recurrences, treatment of underlying predisposing factor, complete source control and appropriate antibiotic therapy are crucial. Antibiotic prophylaxis for recurrent erysipelas and decolonization for MRSA carriers demonstrated some advantages, but also long-term loss of efficacy and possible adverse effects. Clinical score and patients risk stratification could be useful tools to target prophylaxis and decolonization strategies. To reduce hospitalization rates and costs, outpatient oral and parenteral antibiotic therapy (OPAT) and long-acting antibiotics are being implemented.

**Summary:**

Management of RSSTIs requires both preventive interventions on modifiable risk factors and pharmacological strategies, with a patient tailored approach.

## INTRODUCTION

Skin and soft tissue infections (SSTIs) are common infections in both adults and children, both community and hospital acquired. Their incidence varies according with study population, region and definitions applied, but has been increasing in years [1]. Incidence in paediatric patients raised from 23.2 to 62.7/100 000 person years from 2000 to 2006. [2]. For adult patients, incidence has been reported from 49.6 to 77.5/1000 person years [3^▪▪^,^4^,^5^].

Due to the wide variety of clinical entities included under the term of SSTIs, different classifications were proposed. In 2013, the US Food and Drug Administration (FDA) developed the definition of acute bacterial skin and skin-structure infection (ABSSSI), which includes cellulitis/erysipelas, wound infections and major cutaneous abscesses [6], in order to better distinguish complicated SSTIs (cSSTIs). The 2014 Infectious Disease Society of America (IDSA) guidelines proposed a practical classification based on a clinical evaluation (mild/moderate/severe) and distinction between purulent (furuncles, carbuncles, abscesses) and nonpurulent (cellulitis, erysipelas, fasciitis) infections [7].

According with SSTIs severity, associated morbidity and mortality rates may vary. The rates of mortality in patients with SSTIs are generally low, except for necrotizing infections where they can reach up to 70% [7]. As for morbidity, recurrent SSTIs (RSSTIs) are an increasing reported complication, with rates ranging from 7% up to 45% [3^▪▪^,^7^,8^▪^], probably favoured by ageing and comorbidity prevalence among population.

Definitions for RSSTIs are not fully identified. As defined for other types of infections, we consider a recurrent episode the presence of signs and symptoms of infection after an initial resolution or improvement and after a full course of antibiotic therapy. This entity represents a challenge for the clinicians, indeed, they imply multiple courses of antibiotic therapy with the risk of selecting for antimicrobial resistance, side effects related to chronic antibiotic exposure (i.e. *Clostridioides difficile* infection, invasive candidiasis, altered renal and/or liver functions), and prolonged in-hospital stay and/or use of healthcare resources with increased costs.

Our purpose is to summarize the most recent data on RSSTIs risk factors and management. 

**Box 1:**
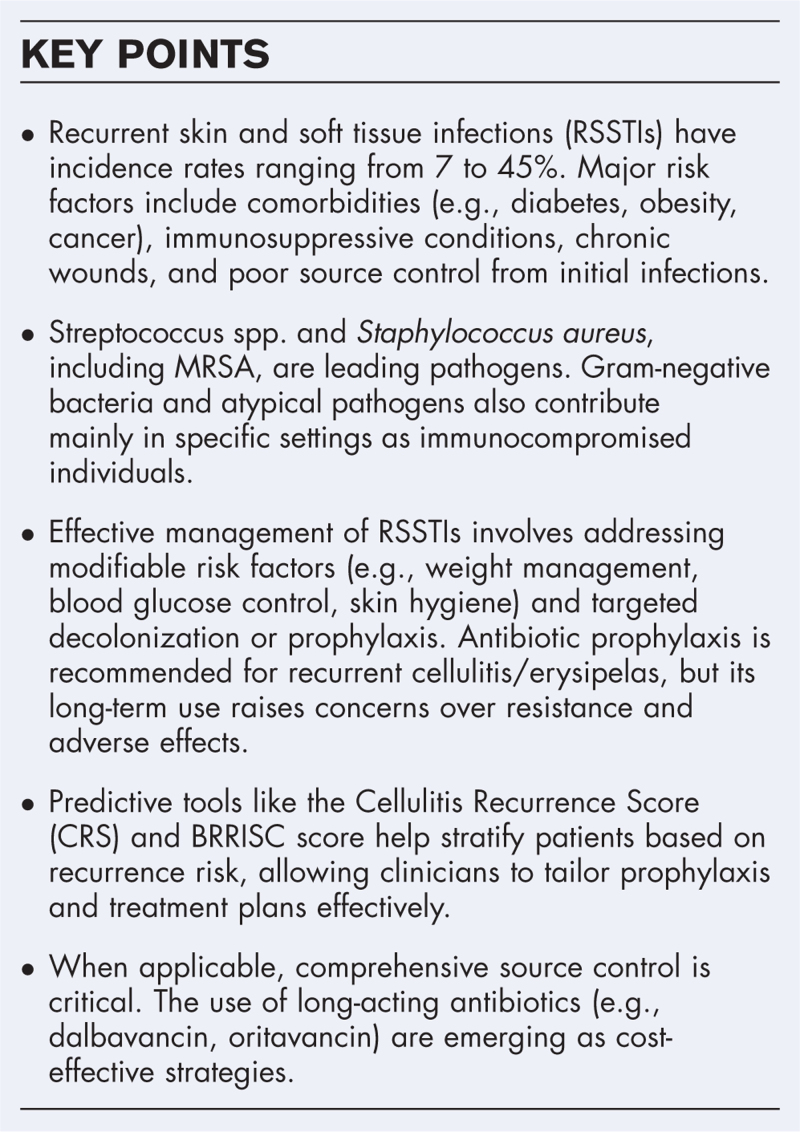
no caption available

## GENERAL RISK FACTORS FOR RECURRENT SKIN AND SOFT TISSUE INFECTIONS

RSSTIs can be the result of a combination of local skin and vascular alteration, patient comorbidities, microbiological factors and/or inappropriate management of the index episode.

In the first place, appropriate initial treatment (medical and/or surgical) is crucial to a prompt resolution of SSTI [9]. Purulent infections usually need incision and complete drainage to resolve. Incomplete source control is the first risk factor for recurrence of purulent infections.

Relevant local factors are venous insufficiency and lymphatic oedema, with poor circulation and impaired vascular-lymphatic return leading to chronic dermatitis, ulcers or wounds. These conditions can facilitate disruption of the skin barrier favouring microorganism penetration and proliferation. Moreover, in case of vascular arteriopathies, such as peripheral chronic occlusive arterial disease, the antibiotics penetration can be slightly reduced as well as achievement of appropriate concentration in the site of infection, with consequent incomplete recovery of a first infective episode.

Comorbidities also concur to the risk of RSSTIs, first of all obesity, diabetes mellitus, cancer and cancer treatment (i.e. chemotherapies and radiations) [10]. Patients with cancer can have a fourfold risk of recurrence of cellulitis. The main risk factors are lymphatic stasis and oedema due to tumour invasion or ab-extrinsic compression, lymph node resection (i.e. in breast cancer surgery) and radiation [11]. Systemic chemotherapy and frequent hospitalization can favour microbiome dysbiosis, colonization with multidrug-resistant organisms (MDRO) and a higher risk of MDRO infections. Among comorbidities, also primary and secondary immunosuppressive conditions are well known risk factors for recurrent infections in general and RSSTIs in particular. Indeed, these infections are particularly common in this setting, due to the loss of barrier integrity, as a consequence of surgical intervention, foreign devices and chronic immunosuppressive therapy. Furthermore, in immunocompromised patients, opportunistic pathogens can be involved as atypical bacteria, mycobacteria, parasites and fungi, as well as atypical localizations are more common than in the nonimmunocompromised patients [12]. As a result, in immunocompromised hosts with RSSTIs, clinical presentation could be nonspecific in relation to the poor immunological condition and response. Tissue sampling is strongly encouraged, whenever possible, in order to obtain a microbiological and histological diagnosis and eventually provide differential diagnosis with other conditions (malignancy infiltration, auto-immune disorders, therapy related skin disorders). Finally, immunological conditions affecting neutrophil activation and effectiveness, such as chronic granulomatous disease (CGD), are predisposing factors for recurrent purulent infections, commonly including SSTIs. Therefore, patients with recurrent purulent infections and abscesses since childhood should undergo evaluation for possible neutrophil disorders [7].

Regarding other host-related factors, people who inject drugs (PWIDs) are particularly susceptible to RSSTIs due to unsafe injection practices and compromised immunity [13^▪^]. SSTIs are the most common infectious complications in PWIDs, with *Streptococcus* and *Staphylococcus* species being the predominant pathogens, but Gram-negative bacteria (GNB) and atypical bacteria are also reported [14].

Finally, microbiological factors strongly contribute to the burden of RSSTIs. Gram-positive cocci (GPC) are the most common pathogen implicated in SSTIs, but GNB and polymicrobial infections are increasingly common. Patients with *Staphylococcus aureus* (*S. aureus*) SSTIs often experience recurrences, affecting between 16 and 19% of healthy adult patients, usually within 3 months from the primary infection [15]. Some *S. aureus* strains harbour intrinsic virulence factors, which can facilitate the recurrence of SSTIs, such as Panton-Valentine Leucocidin (PVL) toxin, which is strongly associated with recurrent purulent SSTIs. Methicillin-resistant *S. aureus* (MRSA) strains are particularly difficult to eradicate, because of the risk of inappropriate antibiotic treatment and chronic colonization of skin and mucosal surface. Indeed, MRSA carriage has been associated with recurrence. Finally, MRSA related to clone USA300 was shown to be responsible for an epidemic spread of invasive infections leading to an increase in the number of individuals with recurrent superficial skin abscesses. This clone was also associated with rising emergence of community-onset MRSA (CO-MRSA) infections. Moreover, highly virulent strains of methicillin-susceptible *S. aureus* (MSSA) belonging to the same genetic lineage as USA300 have been also reported [16,17^▪^,^18^].

*Streptococcus spp.* are frequently cause of RSSTIs, particularly *Streptococcus pyogenes*, also known as Group A *Streptococcus* (GAS), a common colonizer of throat and skin, which can cause mild superficial skin infections (impetigo, erysipelas) as well as invasive life-threatening deep infections (bacteraemia, pneumoniae and necrotizing fasciitis) [19]. A strong association has been described between different types of M surface protein and streptococcal tissue tropism. In a recent review, four emmtypes were defined as “generalist” clusters, frequently isolated from skin, throat and invasive infections (emm89, emm44, emm75 and emm4), while emm53 and emm76 were leading invasive isolates with skin tropism and emm71, emm74, emm55 and emm97 were identified as skin-associated emm-types [20^▪▪^].

GNB are increasingly recognized as important pathogens of SSTIs, particularly in comorbid and immunocompromised patients. Common GNB-causing SSTIs include *Enterobacterales*, *Pseudomonas aeruginosa* and *Acinetobacter baumannii*[20^▪▪^]. Risk factors for GNB SSTIs are recent hospitalization, prior antibiotic use and underlying comorbidities [21–23]. GNB SSTIs and polymicrobial episodes were found at a high risk for initial inappropriate treatment, often associated with MDRO isolation, with a consequent increased risk of new exacerbation and recurrence [22,23].

Other less common pathogen implicated in SSTIs are atypical bacteria, as nontuberculous mycobacteria (NTM). Two main risk factors are contributing to the increased prevalence of these pathogens in SSTIs: the increasing number of immunocompromising conditions and of cosmetic and body-modifying procedures [24]. Common NTM species implicated in SSTIs include rapidly growing mycobacteria, *M. marinum* and *M. avium complex*, with *M. kansasii* also causing SSTIs and osteomyelitis in rare cases. A challenging diagnosis, involving tissue biopsy and molecular tests, and treatment, often requiring both surgical debridement and combination antibiotic therapy, explains the frequent subacute, remitting/recurring course of these SSTIs [25].

## RECURRENT CELLULITIS

The lack of gold standard criteria for the diagnosis of recurrent cellulitis can lead to frequent misdiagnosis with other conditions which usually coexist with and favour SSTIs, such as chronic lymphedema. IDSA guidelines define recurrent cellulitis as the occurrence of 3–4 episodes per year, whereas other international societies define it as the presence of two or more episodes per year, or even as “frequent infections” [7, 26]. On this premise, recurrent cellulitis occurs in approximately 14% within 1 year and 45% of cases within 3 years [26]. Local conditions predisposing to recurrent cellulitis are previous infection in the same site, lower limbs localization, chronic oedema, dermatitis, dermatomycosis, peripheral vascular disease, venous insufficiency or thrombosis, trauma, previous surgery, chronic wounds ulcer, presence of foreign bodies. The most relevant systemic conditions are obesity, diabetes and cancer [8^▪^].

Predictive scores were developed to identify patients with highest risk of recurrence. The Cellulitis Recurrence Score (CRS), for recurring lower limb cellulitis, includes chronic venous insufficiency (1 point), ipsilateral deep vein thrombosis (1 point), lymphedema (2 points) and peripheral vascular disease (3 points). A CRS score higher than 2 was associated with a positive predictive value of 83.6% and negative predictive value of 67.5% [27].

The recently developed Baseline Recurrence Risk in Cellulitis score (BRRISC score) individuate patients at risk of recurrent cellulitis based on eight variables (age, heart rate, urea, platelets, albumin, previous cellulitis, venous insufficiency and liver disease). Categorizing as low (score 0–1), medium (2–5) and high (6–15) risk, recurrence increased fourfold: 3.2% [95% confidence interval (95% CI): 2.3–4.4], 9.7% (8.7–10.8) and 16.6% (13.3–20.4). Patients at a high risk were further divided into four clinical phenotypes: young, acutely unwell with liver disease; comorbid with previous cellulitis and venous insufficiency; chronic renal disease with severe renal impairment; and acute severe illness, with substantial inflammatory responses [28^▪^].

These risk scores could be helpful in optimizing treatment and targeting preventive strategies for recurrent cellulitis.

## RECURRENT ABSCESSES AND PURULENT INFECTIONS

Recurrent abscesses are quite common, with a rate of 7–14% within 2 months of completed therapy [29,30].

This condition can relate to local risk factors such as pilonidal cyst or foreign material. A diagnosis of hidradenitis suppurativa, an inflammatory chronic condition, should be considered in case of painful recurrent abscesses, distributed around the groin, buttocks, breasts and armpits, leaving deep scars and skin tunnels. Other risk factors are intravenous drug administration, incomplete surgical debridement during the first episode, as well as colonization with *S. aureus* and specific immune disorders (i.e. CGD) as aforementioned.

## PREVENTION AND TREATMENT

Management of recurrent SSTIs includes individuation of predisposing conditions and treatment whenever possible. Nonpharmacological interventions include weight and diabetes control, and patient education regarding skin care [7,31].

Antibiotic prophylaxis is indicated only for recurring nonpurulent SSTIs (i.e. cellulitis and erysipelas), for patients with more than two episodes of recurrence per year. Prophylaxis targets beta-haemolytic Streptococci and drugs of choice are low dose oral phenoxymethylpenicillin (penicillin V), intramuscular benzathine penicillin every 2–4 weeks, or erythromycin in case of penicillin allergy [32–36].

Recently, a Cochrane analysis reviewed the five clinical trials designed to set utility of antibiotic prophylaxis and found a reduction in recurrence by 69% (risk ratio 0.31, 95% CI 0.13–0.72), with an estimated number needed to treat (NNT) of six patients with nonpurulent SSTI to prevent a recurrence [37].

Duration of prophylaxis is not clearly defined, ranging from 4 to 52 weeks, but usually continued for at least 6 months. Since the protective effect is lost after discontinuation, a personalized approach is suggested, based on frequency of recurrence and persistence of risk factors [7,8^▪^,^25^].

The counterpart of antibiotic prophylaxis is development of antimicrobial resistance, pharmacological side effects and risk of *Clostridioides difficile* infection. In particular, high rates of macrolide resistance are already described in Europe for Streptococcus spp. and cross resistance between macrolide and clindamycin is largely documented [38].

With these premises, the suggested approach is to target prophylaxis only for high-risk patients, selected by clinical scores, reducing antibiotics misuse [27,28^▪^].

In case of recurrent purulent infections and abscesses associated with pilonidal cyst, hidradenitis suppurativa and foreign bodies, surgical debridement and cultures should be performed [7]. After incision and drainage, 7 days of antibiotic course showed higher curative rate and less recurrences than surgery alone, even for noncomplicated abscesses [29].

Regarding MRSA colonization and infections, patient education is the first measure to contain the risk of spread and recurrence of infection. Therefore, hygiene measures are recommended for all colonized patients and household contacts. For patients with *Staphylococcal* RSSTIs, decolonization with intranasal mupirocin (twice daily for 5 days) and chlorhexidine gluconate body washes (daily for 5 days) are suggested, with the extension of the indication also for household contacts. These interventions can be costly to families and effectiveness seems to fail over time [39^▪^]. Recently, a randomized control trial called “HOME2 study” was performed in a population of paediatric MRSA carriers, with the aim of comparing a broad decolonization approach for all the household contacts of MRSA colonized carriers versus a personalized approach, only for household contacts at a high risk of SSTIs (i.e. household contacts who experienced a SSTI during the study period). The study reached the noninferiority criteria for the personalized approach, which was as effective as the universal decolonization in preventing SSTI. At multivariable analysis, risk factors for cumulative SSTIs were previous episodes of SSTI within 1 year and baseline MRSA colonization of the household contacts [40]. New preventive perspectives are anti-Staphylococcal and anti-Streptococcal group A vaccines. Whereas the former have provided controversial results [41,42^▪^], the latter are still under development [43].

Antibiotic treatment indications for recurrent STTIs do not differ from treatment of the first episode (Table 1); however, empiric initial therapy should be targeted on previous microbiological isolates, if available, in particular in case of previous MRSA isolation [7].

**Table 1:** Main recurrent skin and soft tissue infections: prevalence, risk factors and management

Type of SSTI	Rate of recurrence	Risk factors for recurrence	Prevention	Treatment
Cellulitis/erisipela	14–45%	• previous infection in the same site • lower limbs localization • chronic oedema, dermatitis • dermatomycosis • peripheral vascular disease • venous insufficiency or thrombosis • trauma • previous surgery • chronic wounds ulcer • presence of foreign bodies • obesity • Diabetes mellitus • Cancer • Immunodepression • PWIDs	Non pharmacological measures: • Wight reduction • Blood glucose level control • Skin care Antibiotic prophylaxis: • daily oral phenoxymethylpenicillin (penicillin V) • intramuscular benzathine penicillin every 2–4 weeks • daily erythromycin in case of penicillin allergy	Beta-lactams with anti-GAS activity for 5–10 days
Abscesses and purulent infections	7–14%	• pilonidal cyst • hidradenitis suppurativa • foreign material • previous incomplete surgical debridement • *S. aureus* colonization • PWIDs • Immunosuppressive conditions (in particular CGD)	Non-pharmacological measures: • hand and environmental hygiene Pharmacological measures: MRSA decolonization for colonized patients and household contacts with • intranasal mupirocin BID for 5 days • daily chlorhexidine gluconate body washes for 5 days	Mild Infection: • Incision and drainage (I&D) Moderate/Severe: • Surgical debridement + systemic antibiotic therapy (covering MRSA) for 7 days

BID, bis in die; CGD, chronic granulomatous disease; GAS, Group-A *Streptococcus*; MRSA, methicillin-resistant *Staphylococcus aureus*; PWIDs, people who inject drugs; RSSTIs, recurrent skin and soft tissue infections.

In order to avoid recurrent hospitalization for parenteral antibiotic course, RSSTIs are common indications for outpatient parenteral antibiotic therapy (OPAT). A randomized control trial showed no difference in terms of outcome, between in-hospital versus at home parenteral antibiotic therapy [44]. Moreover, the approval for SSTIs treatment of long-acting semisynthetic glycopeptides with antigram-positive activity (dalbavancin, oritavancin) may offer valid alternatives in the management of RSSTIs. Current national guidelines [7,45] still do not include evidence on the implementation of OPAT with these drugs, however a growing literature supports their use, even in the paediatric population [46,47,48^▪^]. A recent systematic review, network-metanalyses and costs analysis compared standard of care (SOC) for cSSTIs (mainly vancomycin, otherwise linezolid, tedizolid, daptomycin, clindamycin, trimethoprim/sulfamethoxazole, doxycycline, oxacillin, cefazolin, ceftaroline and tigecycline) with telavancin, dalbavancin and oritavancin as treatment for cSSTI. Network meta-analysis showed that clinical response was similar to standard of care [odds ratio (OR) 1.09, 95% CI 0.90–1.33; OR 0.78, 95% CI 0.52–1.18; and OR 1.06, 95% CI 0.85–1.33, respectively]. Costs analysis demonstrated that dalbavancin and oritavancin were less costly compared to other anti-MRSA active antibiotics [49]. Repeated administration of dalbavancin was also shown to be successful in preventing recurrence of cellulitis recurrence in case series [50]. In this regard, follow up of patients treated with long-acting antibiotics should be implemented with therapeutic drug monitoring (TDM) of plasmatic levels, since attaining pharmacokinetic/pharmacodynamic (PK/PD) target of antibiotics is associated with better outcome, lower antimicrobial resistance and lower infection recurrence [51].

## CONCLUSION

RSSTIs are common but challenging clinical entities, with an important burden in terms of morbidity and healthcare-related costs. Recognizing modifiable risk factors and applying both pharmacological and nonpharmacological preventive strategies, along with a tailored approach, are effective in reducing the incidence of these conditions. A growing amount of new therapeutic options, both oral and parenteral, and the chance of out-patients administration are probably meant to change RSSTIs management in the near future.

## Acknowledgements

*None.*

### Financial support and sponsorship

*None.*

### Conflicts of interest

*There are no conflicts of interest.*

## REFERENCES AND RECOMMENDED READING

Papers of particular interest, published within the annual period of review, have been highlighted as:

▪ of special interest
▪▪ of outstanding interest
